# (*E*)-Ethyl 2-(3-cinnamoyl­thio­ureido)acetate

**DOI:** 10.1107/S1600536810037918

**Published:** 2010-10-09

**Authors:** Ibrahim N. Hassan, Bohari M. Yamin, Mohammad B. Kassim

**Affiliations:** a School of Chemical Sciences and Food Technology, Faculty of Science and Technology, Universiti Kebangsaan Malaysia, UKM 43600 Bangi Selangor, Malaysia

## Abstract

In the title compound, C_14_H_16_N_2_O_3_S, the phenyl ring and the ethyl 2-(3-formyl­thio­ureido)acetate fragment adopt an *E* configuration with respect to the C=C bond. An intra­molecular N—H⋯O hydrogen bond generating an *S*(6) ring motif is observed. In the crystal, mol­ecules are linked by N—H⋯S, C—H⋯S and C—H⋯O hydrogen bonds, forming sheets lying parallel to the *ab* plane.

## Related literature

For bond-length data, see: Allen *et al.* (1987 ▶). For related structures, see: Yamin & Hassan (2004 ▶); Hassan *et al.* (2008*a* ▶,*b* ▶,*c* ▶, 2009 ▶); Hung *et al.* (2010 ▶). For the synthesis, see: Hassan *et al.* (2008*a* ▶).
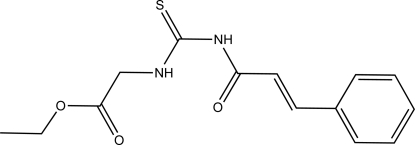


## Experimental

### 

#### Crystal data

C_14_H_16_N_2_O_3_S
*M* *_r_* = 292.35
Orthorhombic, 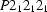

*a* = 5.1867 (9) Å
*b* = 9.7417 (16) Å
*c* = 29.154 (5) Å
*V* = 1473.1 (4) Å^3^
*Z* = 4
Mo *K*α radiation
μ = 0.23 mm^−1^
*T* = 298 K
0.49 × 0.38 × 0.24 mm

#### Data collection

Bruker SMART APEX CCD area-detector diffractometer
Absorption correction: multi-scan (*SADABS*; Sheldrick, 2000 ▶) *T*_min_ = 0.897, *T*_max_ = 0.947
10938 measured reflections
3637 independent reflections
2747 reflections with *I* > 2σ(*I*)
*R*_int_ = 0.033

#### Refinement

*R*[*F* ^2^ > 2σ(*F* ^2^)] = 0.063
*wR*(*F* ^2^) = 0.164
*S* = 1.03
3637 reflections
182 parameters
H-atom parameters constrained
Δρ_max_ = 0.35 e Å^−3^
Δρ_min_ = −0.21 e Å^−3^
Absolute structure: Flack (1983 ▶), 1497 Friedel pairs
Flack parameter: −0.04 (13)

### 

Data collection: *SMART* (Bruker, 2000 ▶); cell refinement: *SAINT* (Bruker, 2000 ▶); data reduction: *SAINT*; program(s) used to solve structure: *SHELXS97* (Sheldrick, 2008 ▶); program(s) used to refine structure: *SHELXL97* (Sheldrick, 2008 ▶); molecular graphics: *SHELXTL* (Sheldrick, 2008 ▶); software used to prepare material for publication: *SHELXTL*, *PARST* (Nardelli, 1995 ▶) and *PLATON* (Spek, 2009 ▶).

## Supplementary Material

Crystal structure: contains datablocks global, I. DOI: 10.1107/S1600536810037918/ci5178sup1.cif

Structure factors: contains datablocks I. DOI: 10.1107/S1600536810037918/ci5178Isup2.hkl

Additional supplementary materials:  crystallographic information; 3D view; checkCIF report

## Figures and Tables

**Table 1:** Hydrogen-bond geometry (Å, °)

*D*—H⋯*A*	*D*—H	H⋯*A*	*D*⋯*A*	*D*—H⋯*A*
N1—H1*A*⋯S1^i^	0.86	2.79	3.631 (2)	166
N2—H2*A*⋯O1	0.86	1.92	2.611 (4)	137
C4—H4*A*⋯O3^ii^	0.93	2.54	3.457 (4)	170
C8—H8*A*⋯S1^i^	0.93	2.86	3.716 (3)	153

Symmetry codes: (i) 
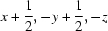
; (ii) 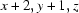.

**Fig. 1.**
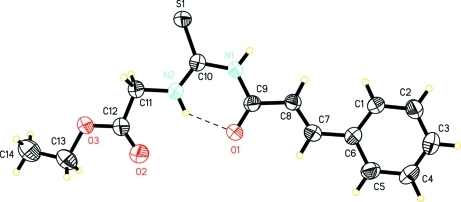
The molecular structure of the title compound, with displacement ellipsoids shown at the 50% probability level.

**Fig. 2.**
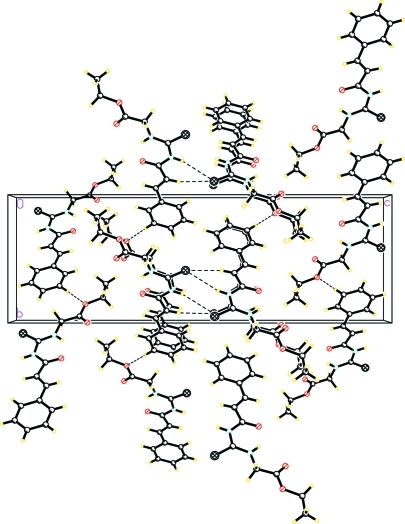
A packing diagram of the title compound, viewed down the a axis. Hydrogen bonds are shown as dashed lines.

## supplementary crystallographic information

### Comment

The title compound, I, is an ethyl ester derivative of glycine thiourea analogoue to our previously reported molecules, ethyl-2-(3- benzoylthioureido)acetate (II) (Hassan *et al.*, 2008*a*). As in most carbonylthiourea derivatives of the type *R*^1^C(O)NHC(S)NH*R*^2^, such as in methyl-2-(3-benzoylthioureido)acetate (Hassan *et al.*, 2009), propyl-2-(3-benzoylthioureido)acetate (Hassan *et al.*, 2008*b*), butyl-2-(3-benzoylthioureido)acetate (Hassan *et al.*, 2008*c*) and 1-(2-morpholinoethyl)-3-(3-phenylacryloyl)thiourea (Yamin & Hassan, 2004), the molecule maintains its *E—Z* configuration with respect to the positions of the cinnamoyl and ethyl acetate groups, respectively, relative to the S atom across the C10—N2 bond (Fig 1). Bond lengths and angles in the molecule are in normal ranges (Allen *et al.*, 1987) and comparable to those observed in (II). However, the C═S bond length [1.675 (3) Å] is slightly longer than that of (II) [1.666 Å]. The cinnamoylthiourea fragment, [S1/O1/N1/N2/C1-C11, A], is essentially planar with a maximum deviation of 0.079 (3) %A, for the atom C1. In the ethyl acetate moeity, [O2/O3/N2/C11-C13, B], the maximum deviation from the mean plane is 0.007 (3) %A for the atom C13. The phenyl ring [C1–C6, C] is inclined to the ethyl acetate mean plane with a dihedral angle of 13.9 (2)° which is larger than that observed in compound (II) [3.6 (1)°]. The additional CH_2_ group introduced a more steric geometry to the ethyl acetate moiety. The dihedral angle between the fragments A/B is 10.8 (1)°. There is one intramolecular hydrogen bond, N2—H2A···O1 (Table 1) which resulted in a formation of pseudo-six-membered ring (N2/H2A/O1/C9/N1/C10) (Fig 1). The molecular packing is stablized by N1—H1A···S1, C4—H4A···O3 and C8—H8A···S1 intermolecular hydrogen bonds, which form a sheet parallel to the *ab* plane.

### Experimental

The title compound was synthesized according to a previously reported procedure (Hassan *et al.*, 2008*a*). Single crystals were obtained by slow evaporation of a CH_2_Cl_2_ solution at room temperature (yield 71%).

### Refinement

H atoms were positioned geometrically [N–H = 0.86 Å and C–H = 0.93–0.97 Å] and allowed to ride on their parent atoms, with *U*_iso_(H) = 1.2*U*_eq_(C,N) and 1.5*U*_eq_(C_methyl_).

### Crystal data

**Table:**

C_14_H_16_N_2_O_3_S	*F*(000) = 616
*M**_r_* = 292.35	*D*_x_ = 1.318 Mg m^−^^3^
Orthorhombic, *P*2_1_2_1_2_1_	Mo *K*α radiation, λ = 0.71073 Å
Hall symbol: P 2ac 2ab	Cell parameters from 2524 reflections
*a* = 5.1867 (9) Å	θ = 2.2–25.5°
*b* = 9.7417 (16) Å	µ = 0.23 mm^−^^1^
*c* = 29.154 (5) Å	*T* = 298 K
*V* = 1473.1 (4) Å^3^	Block, colourless
*Z* = 4	0.49 × 0.38 × 0.24 mm

### Data collection

**Table:**

Bruker SMART APEX CCD area-detector diffractometer	3637 independent reflections
Radiation source: fine-focus sealed tube	2747 reflections with *I* > 2σ(*I*)
graphite	*R*_int_ = 0.033
ω scan	θ_max_ = 28.3°, θ_min_ = 2.2°
Absorption correction: multi-scan (*SADABS*; Sheldrick, 2000)	*h* = −6→6
*T*_min_ = 0.897, *T*_max_ = 0.947	*k* = −12→13
10938 measured reflections	*l* = −32→38

### Refinement

**Table:**

Refinement on *F*^2^	Secondary atom site location: difference Fourier map
Least-squares matrix: full	Hydrogen site location: inferred from neighbouring sites
*R*[*F*^2^ > 2σ(*F*^2^)] = 0.063	H-atom parameters constrained
*wR*(*F*^2^) = 0.164	*w* = 1/[σ^2^(*F*_o_^2^) + (0.1*P*)^2^] where *P* = (*F*_o_^2^ + 2*F*_c_^2^)/3
*S* = 1.03	(Δ/σ)_max_ = 0.036
3637 reflections	Δρ_max_ = 0.35 e Å^−^^3^
182 parameters	Δρ_min_ = −0.21 e Å^−^^3^
0 restraints	Absolute structure: Flack (1983), 1497 Friedel pairs
Primary atom site location: structure-invariant direct methods	Flack parameter: −0.04 (13)

### Special details

**Table:**

Geometry. All e.s.d.'s (except the e.s.d. in the dihedral angle between two l.s. planes) are estimated using the full covariance matrix. The cell e.s.d.'s are taken into account individually in the estimation of e.s.d.'s in distances, angles and torsion angles; correlations between e.s.d.'s in cell parameters are only used when they are defined by crystal symmetry. An approximate (isotropic) treatment of cell e.s.d.'s is used for estimating e.s.d.'s involving l.s. planes.
Refinement. Refinement of *F*^2^ against ALL reflections. The weighted *R*-factor *wR* and goodness of fit *S* are based on *F*^2^, conventional *R*-factors *R* are based on *F*, with *F* set to zero for negative *F*^2^. The threshold expression of *F*^2^ > σ(*F*^2^) is used only for calculating *R*-factors(gt) *etc*. and is not relevant to the choice of reflections for refinement. *R*-factors based on *F*^2^ are statistically about twice as large as those based on *F*, and *R*- factors based on ALL data will be even larger.

### Fractional atomic coordinates and isotropic or equivalent isotropic displacement parameters (Å^2^)

**Table:**

	*x*	*y*	*z*	*U*_iso_*/*U*_eq_	
S1	−0.18635 (18)	0.08578 (8)	0.03548 (2)	0.0573 (2)	
O1	0.3380 (5)	0.2635 (3)	0.14456 (7)	0.0694 (7)	
O2	0.0044 (6)	−0.0041 (3)	0.20692 (9)	0.0952 (10)	
O3	−0.3348 (5)	−0.1415 (2)	0.19707 (7)	0.0712 (7)	
N1	0.1857 (5)	0.2377 (2)	0.07184 (7)	0.0446 (5)	
H1A	0.2047	0.2655	0.0440	0.053*	
N2	−0.0312 (5)	0.0950 (2)	0.12136 (8)	0.0494 (6)	
H2A	0.0690	0.1285	0.1420	0.059*	
C1	0.9341 (6)	0.5968 (3)	0.05420 (11)	0.0540 (7)	
H1B	0.8379	0.5558	0.0310	0.065*	
C2	1.1166 (7)	0.6944 (3)	0.04298 (12)	0.0630 (9)	
H2B	1.1426	0.7182	0.0124	0.076*	
C3	1.2592 (6)	0.7563 (3)	0.07652 (12)	0.0635 (9)	
H3A	1.3788	0.8236	0.0689	0.076*	
C4	1.2251 (7)	0.7186 (3)	0.12129 (13)	0.0661 (9)	
H4A	1.3249	0.7593	0.1441	0.079*	
C5	1.0440 (7)	0.6208 (3)	0.13305 (11)	0.0581 (8)	
H5A	1.0234	0.5962	0.1637	0.070*	
C6	0.8916 (5)	0.5586 (3)	0.09962 (10)	0.0450 (6)	
C7	0.7010 (6)	0.4568 (3)	0.11298 (10)	0.0487 (6)	
H7A	0.6949	0.4335	0.1439	0.058*	
C8	0.5352 (6)	0.3939 (3)	0.08554 (10)	0.0451 (6)	
H8A	0.5372	0.4125	0.0543	0.054*	
C9	0.3480 (6)	0.2950 (3)	0.10411 (9)	0.0465 (6)	
C10	−0.0050 (6)	0.1404 (3)	0.07938 (10)	0.0439 (6)	
C11	−0.2162 (6)	−0.0072 (3)	0.13555 (10)	0.0514 (7)	
H11A	−0.2033	−0.0869	0.1158	0.062*	
H11B	−0.3892	0.0297	0.1328	0.062*	
C12	−0.1669 (7)	−0.0482 (3)	0.18390 (11)	0.0604 (8)	
C13	−0.3116 (12)	−0.1938 (5)	0.24386 (13)	0.1039 (16)	
H13A	−0.1398	−0.2299	0.2490	0.125*	
H13B	−0.3433	−0.1210	0.2658	0.125*	
C14	−0.5011 (16)	−0.3012 (7)	0.24893 (19)	0.165 (3)	
H14A	−0.4645	−0.3534	0.2761	0.247*	
H14B	−0.4953	−0.3606	0.2227	0.247*	
H14C	−0.6697	−0.2612	0.2514	0.247*	

### Atomic displacement parameters (Å^2^)

**Table:**

	*U*^11^	*U*^22^	*U*^33^	*U*^12^	*U*^13^	*U*^23^
S1	0.0656 (5)	0.0633 (4)	0.0430 (4)	−0.0139 (4)	−0.0054 (4)	−0.0001 (3)
O1	0.0813 (16)	0.0836 (14)	0.0431 (11)	−0.0360 (15)	−0.0067 (11)	0.0109 (10)
O2	0.105 (2)	0.113 (2)	0.0677 (17)	−0.049 (2)	−0.0235 (16)	0.0243 (17)
O3	0.0789 (16)	0.0854 (15)	0.0494 (12)	−0.0338 (14)	−0.0044 (12)	0.0145 (11)
N1	0.0475 (12)	0.0476 (11)	0.0386 (11)	−0.0068 (12)	0.0019 (11)	0.0020 (9)
N2	0.0518 (14)	0.0541 (13)	0.0422 (12)	−0.0123 (12)	−0.0024 (10)	0.0020 (11)
C1	0.0510 (17)	0.0602 (16)	0.0509 (16)	−0.0087 (15)	−0.0081 (13)	0.0023 (14)
C2	0.063 (2)	0.0684 (18)	0.0579 (19)	−0.0092 (17)	0.0024 (15)	0.0120 (15)
C3	0.053 (2)	0.0544 (16)	0.084 (2)	−0.0068 (15)	0.0053 (16)	−0.0015 (16)
C4	0.059 (2)	0.0655 (19)	0.073 (2)	−0.0121 (16)	−0.0086 (17)	−0.0198 (17)
C5	0.0598 (19)	0.0647 (19)	0.0497 (17)	−0.0059 (17)	−0.0031 (14)	−0.0092 (14)
C6	0.0396 (14)	0.0439 (14)	0.0513 (15)	0.0033 (11)	−0.0010 (12)	−0.0043 (11)
C7	0.0519 (16)	0.0515 (14)	0.0427 (14)	0.0005 (14)	0.0013 (13)	0.0001 (11)
C8	0.0464 (15)	0.0455 (14)	0.0433 (14)	−0.0002 (13)	−0.0003 (12)	0.0017 (11)
C9	0.0473 (16)	0.0472 (14)	0.0449 (14)	−0.0019 (13)	−0.0044 (12)	0.0012 (11)
C10	0.0431 (16)	0.0393 (12)	0.0492 (15)	0.0014 (12)	−0.0004 (12)	−0.0028 (11)
C11	0.0502 (16)	0.0538 (15)	0.0503 (15)	−0.0080 (14)	−0.0016 (13)	0.0065 (12)
C12	0.0631 (19)	0.0642 (18)	0.0538 (17)	−0.0143 (18)	0.0004 (17)	0.0043 (13)
C13	0.124 (4)	0.125 (3)	0.062 (2)	−0.041 (4)	−0.012 (3)	0.038 (2)
C14	0.184 (6)	0.229 (8)	0.083 (3)	−0.108 (6)	−0.038 (4)	0.083 (4)

### Geometric parameters (Å, °)

**Table:**

S1—C10	1.675 (3)	C4—C5	1.381 (5)
O1—C9	1.219 (3)	C4—H4A	0.93
O2—C12	1.193 (4)	C5—C6	1.393 (4)
O3—C12	1.316 (4)	C5—H5A	0.93
O3—C13	1.462 (4)	C6—C7	1.453 (4)
N1—C9	1.380 (4)	C7—C8	1.325 (4)
N1—C10	1.387 (4)	C7—H7A	0.93
N1—H1A	0.86	C8—C9	1.472 (4)
N2—C10	1.308 (4)	C8—H8A	0.93
N2—C11	1.443 (4)	C11—C12	1.487 (4)
N2—H2A	0.86	C11—H11A	0.97
C1—C2	1.381 (4)	C11—H11B	0.97
C1—C6	1.393 (4)	C13—C14	1.443 (7)
C1—H1B	0.93	C13—H13A	0.97
C2—C3	1.366 (5)	C13—H13B	0.97
C2—H2B	0.93	C14—H14A	0.96
C3—C4	1.367 (5)	C14—H14B	0.96
C3—H3A	0.93	C14—H14C	0.96
			
C12—O3—C13	117.3 (3)	C7—C8—H8A	119.7
C9—N1—C10	127.1 (2)	C9—C8—H8A	119.7
C9—N1—H1A	116.5	O1—C9—N1	122.2 (3)
C10—N1—H1A	116.5	O1—C9—C8	123.2 (3)
C10—N2—C11	124.8 (2)	N1—C9—C8	114.6 (2)
C10—N2—H2A	117.6	N2—C10—N1	117.0 (2)
C11—N2—H2A	117.6	N2—C10—S1	123.3 (2)
C2—C1—C6	121.2 (3)	N1—C10—S1	119.7 (2)
C2—C1—H1B	119.4	N2—C11—C12	110.0 (3)
C6—C1—H1B	119.4	N2—C11—H11A	109.6
C3—C2—C1	120.3 (3)	C12—C11—H11A	109.6
C3—C2—H2B	119.8	N2—C11—H11B	109.7
C1—C2—H2B	119.8	C12—C11—H11B	109.7
C2—C3—C4	119.7 (3)	H11A—C11—H11B	108.2
C2—C3—H3A	120.2	O2—C12—O3	125.3 (3)
C4—C3—H3A	120.2	O2—C12—C11	124.3 (3)
C3—C4—C5	120.6 (3)	O3—C12—C11	110.4 (3)
C3—C4—H4A	119.7	C14—C13—O3	107.0 (4)
C5—C4—H4A	119.7	C14—C13—H13A	110.3
C4—C5—C6	120.8 (3)	O3—C13—H13A	110.3
C4—C5—H5A	119.6	C14—C13—H13B	110.3
C6—C5—H5A	119.6	O3—C13—H13B	110.3
C5—C6—C1	117.3 (3)	H13A—C13—H13B	108.6
C5—C6—C7	119.7 (3)	C13—C14—H14A	109.5
C1—C6—C7	123.0 (3)	C13—C14—H14B	109.5
C8—C7—C6	126.5 (3)	H14A—C14—H14B	109.5
C8—C7—H7A	116.7	C13—C14—H14C	109.5
C6—C7—H7A	116.7	H14A—C14—H14C	109.5
C7—C8—C9	120.6 (3)	H14B—C14—H14C	109.5

### Hydrogen-bond geometry (Å, °)

**Table:**

*D*—H···*A*	*D*—H	H···*A*	*D*···*A*	*D*—H···*A*
N1—H1A···S1^i^	0.86	2.79	3.631 (2)	166
N2—H2A···O1	0.86	1.92	2.611 (4)	137
C4—H4A···O3^ii^	0.93	2.54	3.457 (4)	170
C8—H8A···S1^i^	0.93	2.86	3.716 (3)	153

Symmetry codes: (i) *x*+1/2, −*y*+1/2, −*z*;  (ii) *x*+2, *y*+1, *z*.
